# Optical Coherence Tomography (OCT) Biomarkers for Nonsurgical Management of Postvitrectomy Secondary Macular Holes With Intravitreal Triamcinolone Acetonide (1 mg/0.1 mL): A Case Report of Two Cases

**DOI:** 10.1155/crop/4451513

**Published:** 2025-11-20

**Authors:** Rituraj Videkar, Hassan Al Hasid, Mohammad Fazal Kamal, Gangaprasad Amula, Aneez Shaikh

**Affiliations:** ^1^Fakeeh Univ Hospital, Dubai, UAE; ^2^Dubai Hospital, Dubai, UAE; ^3^Al Ahli Hospital, Doha, Qatar; ^4^Mediclinic WellCare Hospital, Dubai, UAE

**Keywords:** case series, OCT biomarker, secondary macular hole, vitrectomy

## Abstract

**Purpose:**

To report optical coherence tomography (OCT) biomarkers for nonsurgical management of secondary macular holes.

**Case Series:**

We report two cases in which postvitrectomy macular holes were managed with preservative-free intravitreal triamcinolone acetonide (1 mg/0.1 mL). All the patients underwent OCT examination before and after injection. Patients 1 and 2 experienced closure of the macular hole. The OCT biomarkers for the patients were (1) an apical diameter of less than 100 microns, (2) the presence of subretinal fluid, (3) the presence of a perifoveal cuff of fluid, and (4) the absence of preretinal traction.

**Discussion:**

The incidence of secondary macular holes is reported to range from 0.24% to 1.9% of patients who undergo vitrectomy. Secondary macular holes are also reported postvitrectomy due to inadvertent iatrogenic trauma, cystoid macular edema, etc. The treatment of secondary macular holes essentially involves various modifications of the ILM flap technique. There have been reports on the closure of secondary macular holes with the intravitreal injection of triamcinolone acetonide, intravitreal bevacizumab, aflibercept, NSAID, and difluprednate. The identification of OCT biomarkers prior to vitrectomy can help in targeted use of nonsurgical treatment modalities in the management of secondary macular holes with the intravitreal injection of triamcinolone acetonide (1 mg/0.1 mL).

**Conclusion:**

Identification of OCT biomarkers can help in the nonsurgical management of secondary macular holes.

## 1. Introduction

Macular holes are relatively uncommon retinal pathologies, with a reported incidence of 0.02% to 0.8% [1]. The understanding of the development, investigation, and management of macular holes has evolved from the time of the first reported traumatic macular hole to the present day [2]. Optical coherence tomography of the macula has played an important role in the classification and management of macular holes. Idiopathic macular holes form due to posterior vitreous detachment induced antero-posterior, perifoveal, and tangential traction with dissolution of the Müller cell cone [3, 4]. The mechanism of secondary macular hole formation is similar; however, the mechanisms differ because of the associations of secondary macular holes with various retinal pathologies. Secondary macular holes contrast with idiopathic macular holes in more than one way. They are associated with sustained cystoid macular edema, cystoid macular degeneration, macular thinning due to ischemia, tangential traction due to a thick ILM, and preretinal membrane formation postvitrectomy [5]. Pathologies such as retinal detachment, high myopia, and the epiretinal membrane are associated pathologies with this entity. The incidence of secondary macular holes is reported to range from 0.24% to 1.9% of patients who undergo vitrectomy [6]. Secondary macular holes are also reported postvitrectomy due to inadvertent iatrogenic trauma. The treatment of secondary macular holes essentially involves various modifications of the ILM flap technique. There have been reports on the closure of secondary macular holes with the intravitreal injection of triamcinolone acetonide, intravitreal bevacizumab, aflibercept, NSAID, and difluprednate. Nonsurgical management of macular hole is not a novel treatment modality and has been discussed in literature [7–10]. In the following case series, we describe the OCT biomarkers which aided in nonsurgical management patients with secondary macular holes.

## 2. Case Report

### 2.1. Patient 1

**Figure 1:**
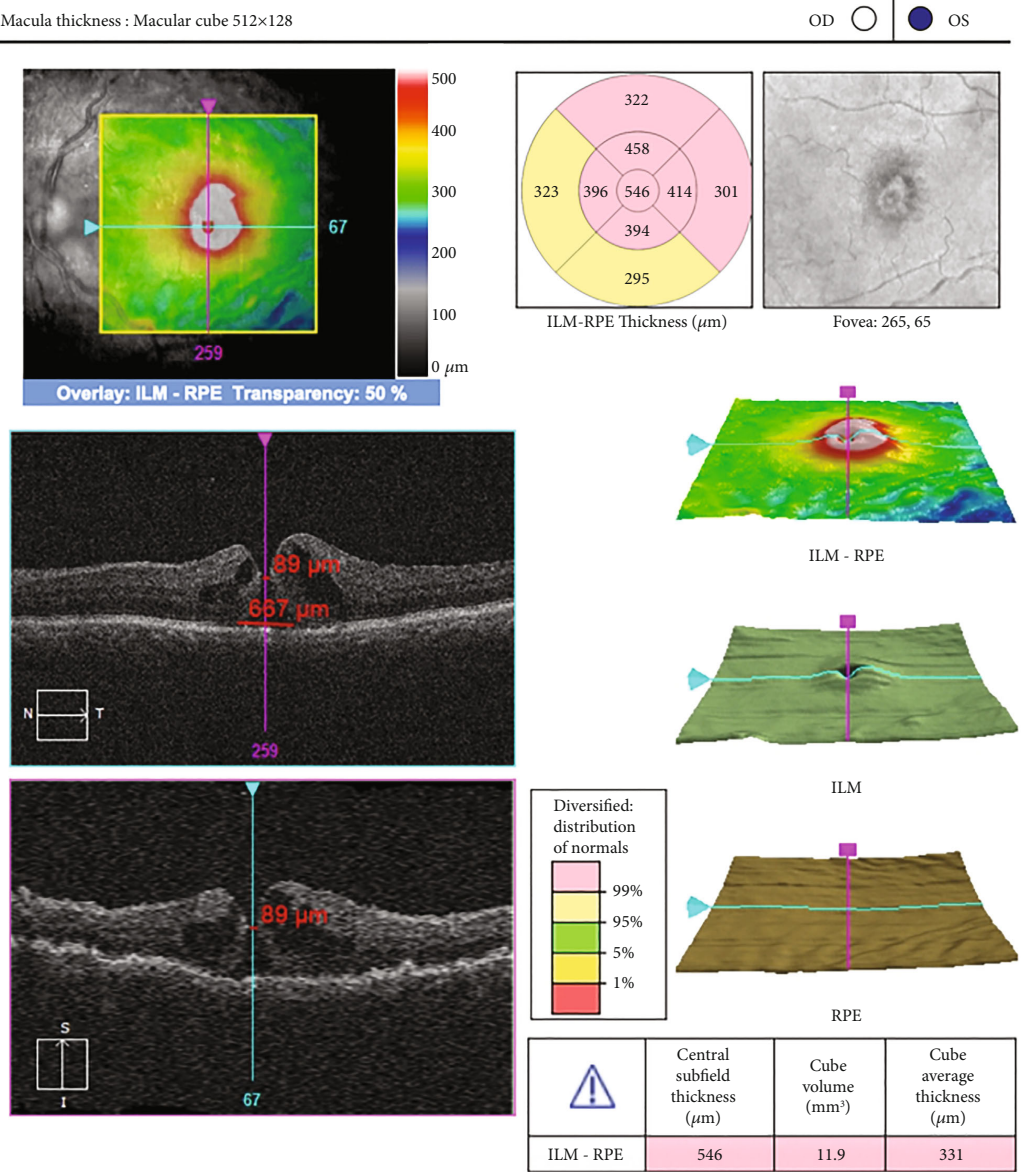
Macular hole with an apical diameter of 89 microns and a basal diameter of 667 microns with the presence of a perifoveal cuff of fluid and subretinal fluid (patient 1).

A 60-year-old woman presented with vision in OD of 0.4, OS of 0.3, and an intraocular pressure (IOP) OU of 16 mm Hg. She had a history of complicated cataract surgery in the left eye, during which she suffered a nucleus drop and subsequently underwent vitrectomy for nuclear fragment removal and intraocular lens implantation. An anterior segment examination of the right eye revealed grade 3 nuclear sclerosis, and the left eye revealed sulcus placement in the posterior chamber of the intraocular lens. The rest of the anterior segment findings for the left eye were normal. On posterior segment examination, the right eye fundus was normal, whereas the left eye revealed a vitrectomized eye with a full-thickness macular hole. The patient had received a course of antibiotic steroid eye drops for 4 weeks after primary surgery. The OCT (Zeiss) macula was performed for the patient's left eye, which was suggestive of a macular hole with an apical diameter of 89 microns and a basal diameter of 667 microns with the presence of a perifoveal cuff of fluid, subretinal fluid (Figure 1), and a flat retina.

**Figure 2:**
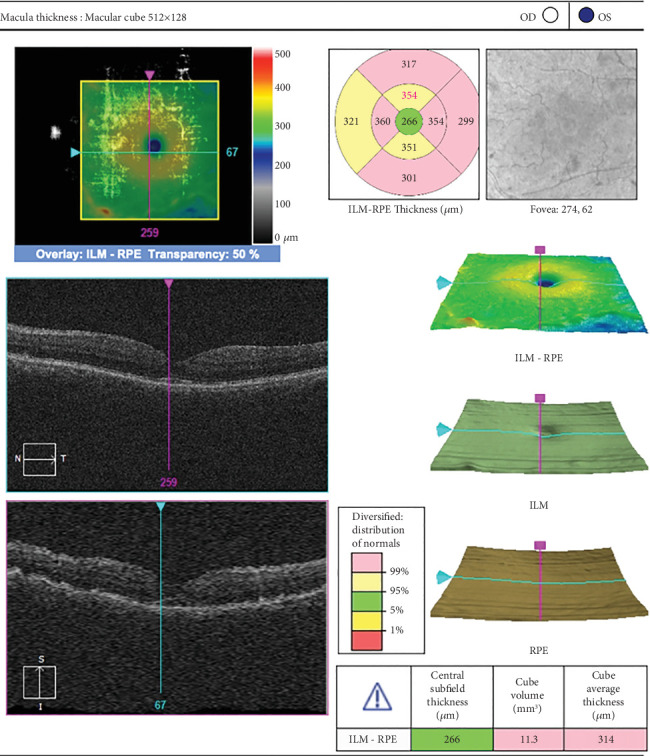
Postinjection OCT was suggestive of a closed macular hole with a central retinal thickness of 266 microns and the presence of some trace subretinal fluid (patient 1).

The patient received preservative-free intravitreal triamcinolone acetonide (1 mg/0.1 mL) for the left eye. Four weeks after the intravitreal injection, her vision in the left eye was 0.3, and her IOP was 18 mm Hg. Anterior segment examination of the left eye revealed pseudophakia, whereas fundus examination revealed a closed macular hole with a flat retina. Postinjection OCT revealed a closed macular hole with a central retinal thickness of 266 microns and the presence of some trace subretinal fluid (Figure 2).

**Figure 3:**
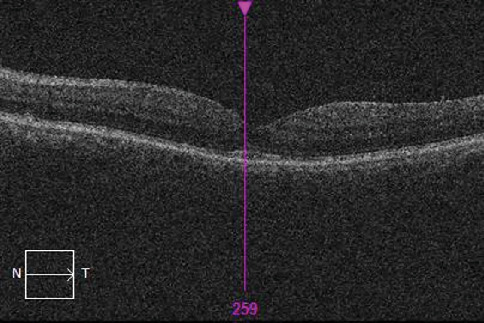
Postinjection OCT image showing a closed macular hole and resolution of subretinal fluid with a central retinal thickness of 250 microns (patient 1).

The patient was requested to continue with prednisolone eye drops of 1% for the next 6 weeks. The follow-up OCT conducted 6 months postinjection revealed a closed macular hole and resolution of subretinal fluid with a central retinal thickness of 250 microns (Figure 3).

### 2.2. Patient 2

**Figure 4:**
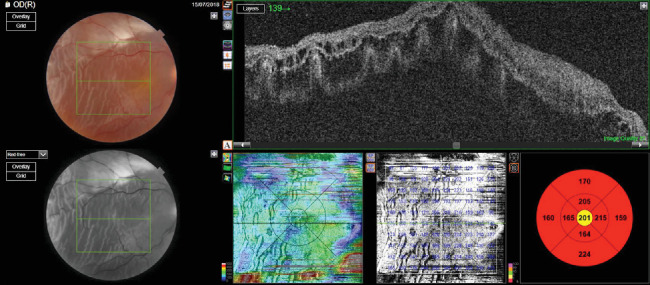
Fundus photo and OCT image showing retinal detachment (patient 2).

A 55-year-old male underwent vitrectomy for retinal detachment in the right eye. The patient had undergone internal limiting membrane peeling at the macula at the time of surgery (Figure 4).

**Figure 5:**
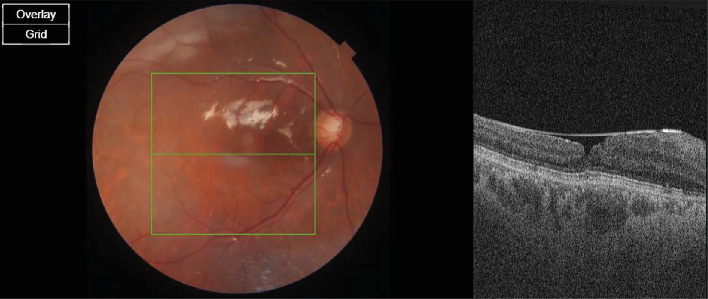
Fundus photo and OCT showing attached retina with silicone oil tamponade (patient 2).

**Figure 6:**
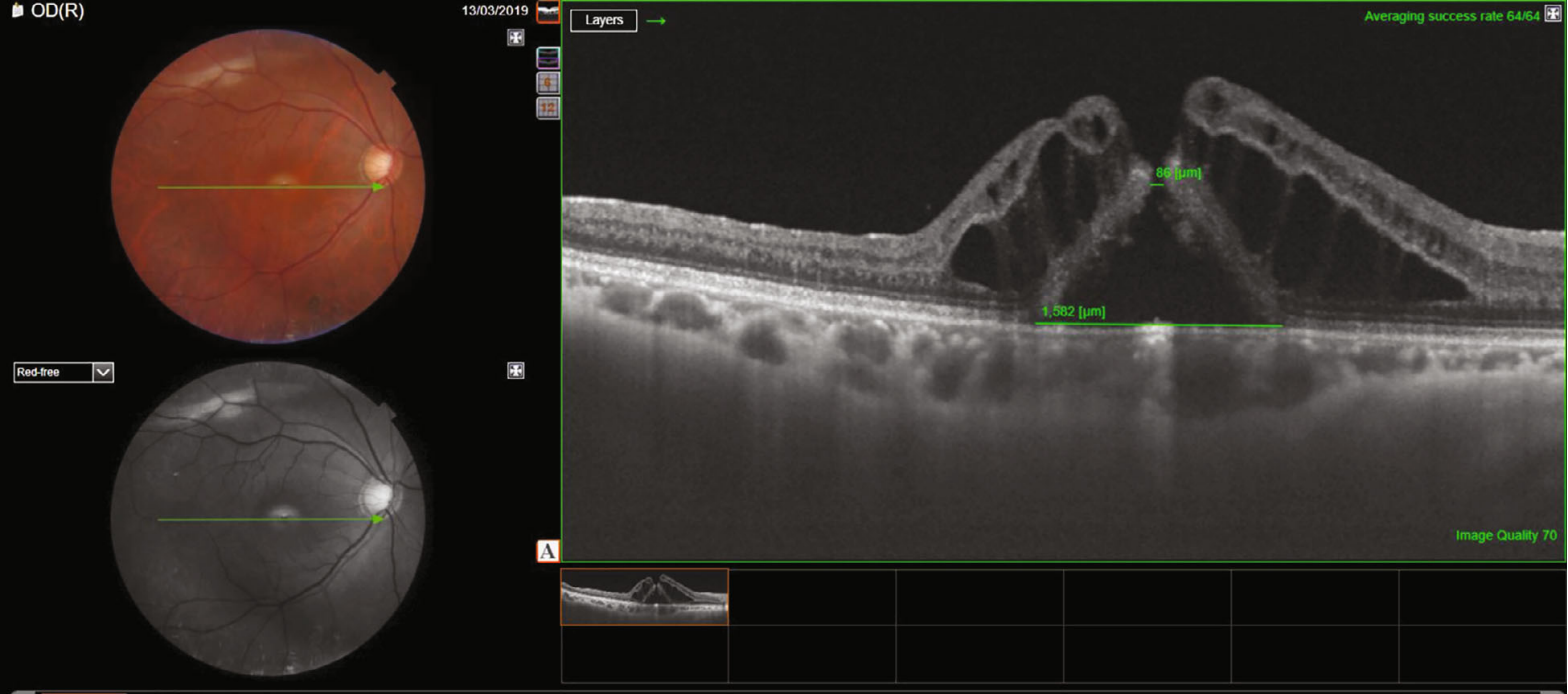
Post oil removal fundus photo and OCT image showing an attached retina with a macular hole with an apical diameter of 86 microns and a basal diameter of 1582 microns (patient 2).

Silicone oil was used as a tamponade agent (Figures 5 and 6).

After vitrectomy, the vision improved to 0.3. The patient underwent phacoemulsification with intraocular lens implantation with removal of silicone oil 12 weeks after the primary surgery. Four weeks after silicone oil removal, the vision of the patient was 0.1 intraocular pressure (IOP) 12 mm Hg. Anterior segment examination of the right eye was suggestive of pseudophakia, whereas the fundus was suggestive of a normal disc with a macular hole (Figure 6).

**Figure 7:**
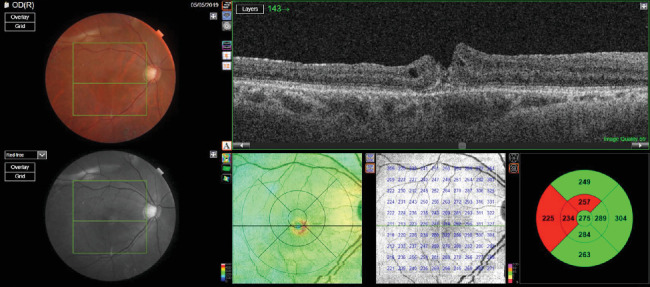
Postinjection OCT image showing a closed macular hole with a central retinal thickness of 275 microns (patient 2).

The patient had received a course of antibiotic and steroid eye drops for 4 weeks after oil removal. The patient underwent OCT macula for the right eye, which was suggestive of a full-thickness macular hole with subretinal and perifoveal cuffs of fluid with a basal diameter of 1582 microns and an apical diameter of 86 mics. The patient received a preservative-free intravitreal injection of triamcinolone acetonide (1 mg/0.1 mL) in the right eye. The patient was reviewed 4 weeks after the injection. The vision of the patient in the right eye improved to 0.3, and the intraocular pressure was 10 mm Hg. Anterior segment examination of the right eye revealed pseudophakia, whereas the fundus was suggestive of a normal disc with a closed macular hole with a flat and attached retina. The postinjection OCT macula for the right eye was suggestive of a closed macular hole with a central retinal thickness of 275 microns (Figure 7).

**Figure 8:**
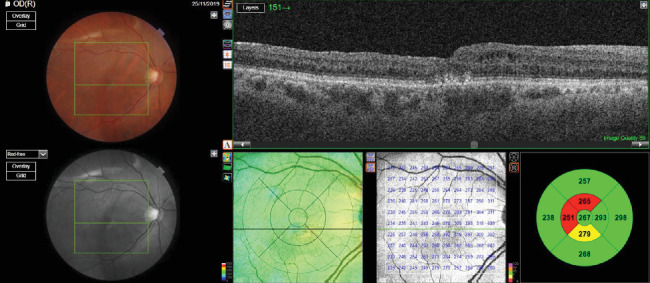
Postinjection OCT at 6 months showing a closed macular hole with a central retinal thickness of 267 microns (patient 2).

OCT, which was performed at the 6-month follow-up postinjection, also revealed a closed macular hole with a central retinal thickness of 267 microns (Figure 8).

## 3. Discussion

The literature mentions the occurrence of secondary macular holes after vitrectomy. The mechanism of macular hole formation is speculated to be related to direct or indirect trauma occurring during vitrectomy [6, 7]. A secondary macular hole forms after the internal limiting membrane is split after PVD induction or postvitrectomy, followed by hydration of the internal retinal layers [11].

In cases of blunt trauma, there is equatorial expansion with a trampoline-like effect on the posterior pole, leading to a macular hole. A similar mechanism can occur during intraocular pressure fluctuations, especially when phacoemulsification is combined with silicone oil removal. The vitreous acts as a dampening agent for shock wave dissipation during blunt trauma [12]. Vitrectomized eyes can be more prone to secondary macular hole formation because of the absence of this cushioning effect.

Cystoid spaces in the retina and neurosensory detachment are known OCT biomarkers of retinal inflammation [13]. In the presence of a preexisting split in the ILM, neurosensory detachment or persistent cystoid macular edema can progress to the development of a small macular hole. Steroid therapy is a bulwark in the management of cystoid macular edema. The mode of delivery can be topical, posterior subtenon, or intravitreal. Triamcinolone acetonide is used to treat cystoid macular edema. Intravitreal triamcinolone acetonide is reported to be a treatment modality for small persistent macular hole [7].

### 3.1. Mechanism of Closure of the Macular Hole

The secondary macular hole in our cases could have been the result of cystoid macular edema post vitrectomy. The presence of a perifoveal cuff of fluid and subretinal fluid indicates the acute course of events leading to the development of the macular hole. The underlying inflammation resolved after intravitreal triamcinolone acetonide injection, resulting in resolution and closure of the macular hole. It can be argued that there could have been spontaneous closure of macular hole; however, this mechanism seems to be unlikely as hole closure was seen within 4 weeks of intravitreal injection of triamcinolone acetonide.

### 3.2. Analysis of the OCT Biomarkers

The OCT biomarkers in the described cases were the presence of perifoveal cuffs of fluid, subretinal fluid, and small macular holes (< 100 microns), along with the absence of a preretinal tractional component.

### 3.3. OCT Biomarkers as Guide for Nonsurgical Management of Secondary Macula Hole

Based on this case series, we hypothesize that a macular hole < 100 microns, the presence of a perifoveal cuff of fluid in both the lateral walls of the macular hole and subretinal fluid, and the absence of a preretinal tractional component can be predictive factors for the closure of secondary macular holes after intravitreal triamcinolone acetonide injection.

The management of postvitrectomy macular holes is essentially surgical and involves ILM peeling and gas tamponade [14, 15]. The visual prognosis depends upon the underlying pathology. A secondary macular hole is usually associated with a poor visual prognosis [16]. OCT biomarkers like size of hole, intraretinal fluid, and subretinal fluid have been discussed in the literature in the context of traumatic macular holes with surgical option being preferred as the choice of treatment. However, the average size of the macular hole in this study was 619 microns [17]. There have been earlier reports of intravitreal use of triamcinolone acetonide for postvitrectomy macular hole. The presumed mechanism of action of macular hole closure being anti-inflammatory activity of triamcinolone and retinal pigment epithelial pumping activity [18, 19]. Although surgical intervention is the accepted mode of treatment for postvitrectomy secondary macular holes, the OCT biomarkers can help in identifying a case group which can possibly benefit with nonsurgical management. We as authors agree that nonsurgical management in the form of intravitreal injection of triamcinolone acetonide 1 mg/0.1 mL for postvitrectomy secondary macular hole is not a novel treatment modality, but elaboration of the OCT biomarkers in the context of nonsurgical management make our case report unique. The OCT biomarkers that we have discussed can help in decision making in such cases. However, we also believe that these OCT biomarkers need to be further validated with large multicentric studies.

## 4. Conclusion

This case report highlights the use of OCT biomarkers for targeted use of steroid therapy as a mode of intervention. Before opting for the surgical approach, which is revitrectomy with a membrane peel, OCT biomarkers of the secondary macular hole can serve as guidelines for nonsurgical management. This will help in providing minimalistic mode of treatment in these eyes.

## Data Availability

The data that supports the findings of the study are available on request from the corresponding author. The data are not publicly available due to privacy or ethical restrictions.
